# The many faces of ubiquitinated histone H2A: insights from the DUBs

**DOI:** 10.1186/1747-1028-3-8

**Published:** 2008-04-22

**Authors:** Joseph HA Vissers, Francesco Nicassio, Maarten van Lohuizen, Pier Paolo Di Fiore, Elisabetta Citterio

**Affiliations:** 1 Division of Molecular Genetics, The Netherlands Cancer Institute, Plesmanlaan 121, 1066 CX Amsterdam, The Netherlands; 2 IFOM, Istituto FIRC di Oncologia Molecolare, Via Adamello 16, 20139, Milan, Italy; 3 Istituto Europeo di Oncologia, Via Ripamonti 435, 20141, Milan, Italy; 4 Dipartimento di Medicina, Chirurgia ed Odontoiatria, Universita' di Milano, 20112, Milan, Italy

## Abstract

Monoubiquitination of H2A is a major histone modification in mammalian cells. Understanding how monoubiquitinated H2A (uH2A) regulates DNA-based processes in the context of chromatin is a challenging question. Work in the past years linked uH2A to transcriptional repression by the Polycomb group proteins of developmental regulators. Recently, a number of mammalian deubiquitinating enzymes (DUBs) that catalyze the removal of ubiquitin from H2A have been discovered. These studies provide convincing evidence that H2A deubiquitination is connected with gene activation. In addition, uH2A regulatory enzymes have crucial roles in the cellular response to DNA damage and in cell cycle progression. In this review we will discuss new insights into uH2A biology, with emphasis on the H2A DUBs.

## Background

Conjugation of ubiquitin (Ub) occurs through the concerted action of an ATP-dependent Ub-activating enzyme (E1), a Ub conjugating enzyme (E2), and a Ub ligase (E3) [1]. The 76-amino acid protein Ub can be conjugated to target proteins in multiple ways, conferring a high potential of diversity to Ub-mediated signaling [2]. As a monomer, Ub can be linked to one (monoubiquitination) or several lysines (multiple monoubiquitination) on target proteins. In addition, Ub has seven lysine residues which can be modified to form polyubiquitin chains. Lysine 48-linked Ub chains generally target proteins for proteolytic destruction. In contrast, monoubiquitination as well as chain formation through lysine 63 have a regulatory role in various processes, including endocytosis, DNA repair, transcription and chromatin regulation [2]. Ubiquitin signaling is transduced by the so called "ubiquitin receptors', proteins which utilize ubiquitin binding domains to interact with ubiquitinated targets [3].

Processes that impact on DNA, such as transcription, DNA replication, DNA repair and mitosis have in common the ability to perform DNA transactions in the chromatin environment. Histones are essential proteins that compact the DNA in the basic unit of chromatin, the nucleosome. In the nucleosome core particle, a tetramer of (H3-H4)2 is flanked by two dimers of H2A-H2B to form the histone octamer, around which 146 bp of DNA is wrapped [4]. Core histones interact with the DNA and with each other through a histone fold domain. In addition, their unstructured N-terminal or C-terminal (in the case of H2A and H2B) "tails" protrude from the nucleosome and provide sites for covalent modification by a variety of enzymes. Histone modifications act both directly (affecting contacts between nucleosomes) and indirectly (through the recruitment of non-histone proteins) in order to orchestrate chromatin environment [5]. It has become clear that covalent modifications ("marks") can influence one another and their combination has been proposed to constitute a "histone code" that regulates chromatin-based processes [6].

Monoubiquitination of histone H2A is one of the most abundant histone modifications in mammalian cells. The ubiquitination site has been mapped to lysine 119 (K119) on the carboxyterminal tail of H2A and monoubiquitinated H2A (uH2A) has been estimated to comprise between 5–15% of H2A. Histone H2B is monoubiquitinated at K120 in about 1% of total H2B [7-10]. Despite H2A being the first protein shown to be ubiquitinated, the function of uH2A has remained obscure for a long time. Only recently, the discovery that the Polycomb protein complex Ring1A/B-Bmi1 is a major E3 ligase targeting H2A, strongly linked uH2A to gene silencing and tumor development [11-14]; reviewed in [15]. Accumulating evidence supports an additional role of uH2A in the maintenance of genome integrity, further highlighting the potential impact of this modification on neoplastic cell growth [16-21].

Ubiquitination of H2A is dynamic, as suggested by the observations that global levels of uH2A vary during the cell cycle [22-25]. Accordingly, the existence of several mammalian H2A de-ubiquitinating enzymes (DUBs) has recently been reported. These include members of two distinct protease families [26]. In particular, 2A-DUB belongs to the JAMM/MPN+ family, while USP3, Ubp-M (USP16), USP21 and USP22 are part of the USP (Ubiquitin Specific Protease) family [17,24,25,27-29] (Table 1). In this review we discuss new findings on how uH2A might regulate transcription, as well as the emerging roles of uH2A in DNA damage signaling and cell cycle progression. The focus will be on the lessons learned from the H2A DUBs.

**Table 1:** Mammalian H2A deubiquitinating enzymes (DUBs)

**DUB**	**Domain structure**	**Substrate**	**Interactors**	**Process**	**Ref.**
USP3	ZnF-UBP, UCH	H2A, H2B	n.d.	DNA damage response; S-phase progression	[17]
USP16/UbpM	ZnF-UBP, UCH	H2A	n.d.	Transcription of HoxD10; G2/M transition	[24,33]
USP21	UCH	H2A	n.d.	Transcription	[25]
USP22	ZnF-UBP, UCH	H2A, H2B	SAGA complex	Transcriptional coactivator with Myc, AR, ER, GR; G1/S transition	[28,29]
2A-DUB/KIAA1915/MYSM1	SANT, SWIRM, JAMM/MPN+	H2A	p/CAF, Trip5/KIF11, Rbm10	Transcriptional coactivator with AR	[27]

Abbreviations: ZnF-UBP, Zinc Finger-Ubiquitin Binding Domain; UCH, Ubiquitin C Terminal Hydrolase domain; AR, Androgen Receptor; ER, Estrogen Receptor; GR, Growth hormone Receptor; 2A-DUB, H2A DeUBiquitinase; JAMM, JAB1/MPN/Mov34 metalloenzyme domain; MPN+, Mpr1, Pad1 N-terminal + domain; p/CAF, p300/CBP Associated Factor; n.d., not determined.

## uH2A in gene repression

The role of uH2A in transcription has been controversial (reviewed in [9,10]). Only recently, the characterization of the Polycomb repressive complex PRC1 as ubiquitin ligase for H2A strongly linked this modification to silencing of developmental control genes and to X-inactivation. Ring1A and Ring1B (RING1 and RNF2 in man), which harbor a RING domain, are the active E3 ligase components of the complex and are responsible for the deposition of bulk monoubiquitinated H2A ([11-14]; reviewed in [15]) (Table 2). In agreement with a repressive role, uH2A levels at Polycomb-repressed promoters decrease in Ring1A/B deficient cells and this is accompanied by an induction of expression of target genes [11,13]. In addition to PRC1, Ring1B has been found in separate repressive complexes containing E2F-6 [30] and the Fbxl10 (JHD1B) and BcoR corepressors [31,32], respectively.

**Table 2:** Mammalian H2A E3 ubiquitin ligases

**E3 ligase**	**Domain structure**	**Substrate**	**Interactors**	**Process**	**Ref.**
Ring1A/RING1, Ring1B/RNF2	RING	H2A	PRC1; E2F-6; Fbxl10/BcoR complexes	Repression of transcription	[11-14, 30-32,86,87]
RNF8	FHA, RING	H2A/H2AX	n.d.	DNA damage response; G2/M transition	[18,19,54,55,63,84,85]
2A-HUB/KIAA0675/hRUL138/DZIP3	RING	H2A	N-CoR, HDAC1, HDAC3	Repression of specific chemokine genes	[40]

Abbreviations: PRC1, Polycomb Repressive Complex 1; Fbxl10, F-box and leucine-rich repeat protein 10 (JHD1B); BcoR, BCL6 co-repressor; RNF8, Ring Finger protein 8; FHA, forkhead-associated phosphopeptide-binding domain; N-CoR, Nuclear receptor Co-Repressor; HDAC, Histone De-Acetylase; n.d., not determined.

Using different approaches, recent work shows that DUBs for uH2A play functional roles in gene activation, providing independent evidence that uH2A antagonizes transcription. In this section we will briefly summarize the findings with respect to gene expression regulation obtained with the H2A DUBs Ubp-M (USP16), USP21, 2A-DUB, and USP22 [24,25,27-29] (Table 1). This will be followed by a more detailed discussion of the potential effects of uH2A on different phases of transcription. Finally, clues to the mechanistic aspects of uH2A-mediated repression that arise from these and other studies will be presented.

### H2A DUBs: novel insights into uH2A-mediated transcription inhibition

#### Ubp-M (USP16)

Extensive biochemical purification of a H2A de-ubiquitinating activity from HeLa cells, identified Ubp-M as the responsible enzyme [24]. In agreement, the *in vitro *activity of Ubp-M towards uH2A had been reported before [33]. By double chromatin immunoprecipitation experiments, in Ubp-M knockdown (KD) HeLa cells, Joo *et al*. showed increased uH2A levels at the promotor of a homeotic (Hox) gene, *HoxD10*, accompanied by gene repression. Wildtype but not catalytic mutant Ubp-M could rescue *HoxD10 *expression [24]. The *in vivo *relevance of Ubp-M mediated Hox gene activation was suggested by the observation that injection of Ubp-M antibodies in *Xenopus *embryos led to deregulation of *HoxD10 *expression and defects in posterior development. This anterior-posterior transformation phenotype is consistent with a Polycomb-antagonistic function (reviewed in [15]). Altogether, these data suggest that Ubp-M counteracts the role of Polycomb proteins in Hox gene repression through H2A deubiquitination.

#### USP21

Nakagawa and colleagues used liver regeneration as a model system [25]. Examining gene expression changes after hepatectomy, the authors found that USP21 is upregulated. As liver regeneration is associated with a decrease in global uH2A, USP21 was hypothesized to target H2A for deubiquitination during this process. USP21 DUB activity towards H2A was confirmed *in vitro*. Evidence for a repressive role of uH2A came from *in vitro *transcription assays, in which chromatin templates reconstituted with uH2A inhibited transcript formation. *In vivo*, overexpression of USP21 in liver correlated with low levels of uH2A and increased expression of a gene, *Serpina6*, which is downregulated during normal hepatocyte regeneration.

#### 2A-DUB

**Figure 1:**
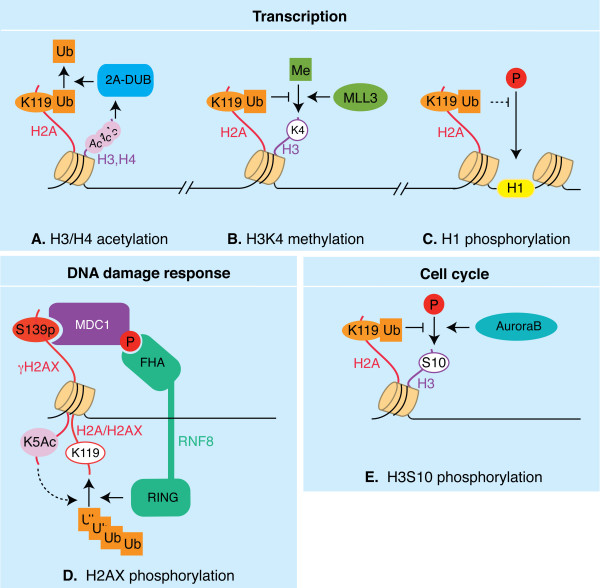
**Crosstalk between monoubiquitinated H2A (uH2A) and other histone modifications**. Functional implications in transcription regulation (A-C), DNA damage response (D) and cell cycle progression (E) are illustrated. **A**. H3/H4 acetylation stimulates de-ubiquitination of uH2A by 2A-DUB *in vitro*, linking these modifications in the regulation of transcription. **B**. uH2A prevents H3K4 methylation by MLL3 *in vitro*. This is possibly one of the mechanisms by which uH2A negatively affects transcription initiation. **C**. Elevated global levels of uH2A correlate with low phosphorylation of linker histone H1, as observed upon knockdown of one of the H2A DUBs, 2A-DUB. Phosphorylation of H1 is thought to favor enhanced chromatin dissociation of this histone. uH2A might, by promoting/stabilizing H1 association with the nucleosome, diminish chromatin dynamics, thereby negatively affecting transcription. **D**. Histone phosphorylation and ubiquitination synergize in DNA damage signaling upon ionizing radiation (IR). Upon IR, phosphorylation of H2AX leads to recruitment and phosphorylation of MDC1. Phosphorylated MDC1 recruits RNF8 through its FHA domain. RNF8 subsequently polyubiquitinates H2A and H2AX. Also, TIP60-dependent acetylation of H2AX on K5 favors H2AX polyubiquitination upon IR. **E**. uH2A inhibits H3 S10 phosphorylation by AuroraB kinase *in vitro*, providing a potential mechanism for regulation of G2/M transition *in vivo*. The labels "Ub", "Ac", "Me" and "P" refer to monoubiquitination, acetylation, di- and trimethylation, and phosphorylation respectively.

2A-DUB was identified as a positive regulator of androgen receptor (AR) transactivation activity on a reporter gene and it was characterized as a histone H2A DUB *in vitro *and *in vivo *[27]. 2A-DUB is unique among the uH2A proteases identified so far, harboring a JAMM/MPN+ domain, and SANT and SWIRM domains, frequently found in DNA and chromatin-associated proteins [26,27]. Knockdown of 2A-DUB resulted in the attenuation of dihydrotestosterone-induced gene expression in a prostate cancer cell line, confirming an AR-coregulatory role for the endogenous protein. 2A-DUB is associated with the p/CAF histone acetyl transferase. This led to the investigation of a possible link between histone acetylation and H2A deubiquitination. It was found that histone acetylation (*in vitro*) and p/CAF (*in vivo*) facilitated H2A de-ubiquitination (Figure 1A). However, p/CAF did not apparently affect 2A-DUB binding with the promoter, suggesting that 2A-DUB is not directly recruited by p/CAF. Instead, p/CAF dependent acetylation may, directly or indirectly, influence 2A-DUB activity.

#### USP22

H2B is the only histone known to be ubiquitinated in *S. cerevisiae *[34]. Monoubiquitinated H2B (uH2B) is highly dynamic and sequential ubiquitination and deubiquitination are required for efficient transcription (reviewed in [10]). A DUB capable of de-ubiquitinating H2B, Ubp8, is present in the yeast SAGA coactivator complex. Two recent papers show that the mammalian counterpart of the SAGA complex contains an Ubp8 homolog, the DUB USP22 [28,29], which is also conserved in *Drosophila *[35]. In addition to H2B, USP22 de-ubiquitinates H2A *in vitro *[29]. As for Ubp8, an activating role of USP22 has been reported. Recruitment of the SAGA complex to target genes is known to be dependent on sequence specific transcription factors [36,37]. Accordingly, Zhang and colleagues showed that USP22 was recruited to myc target genes in a myc-dependent fashion [28].

### Does uH2A affect specific steps of transcription?

RNA polymerase II- mediated transcription can be subdivided in three phases: initiation, elongation and termination. These phases are associated with distinct histone modifications as well as specific phosphorylation patterns of the heptad repeats of the C-terminal domain (CTD) of the largest subunit of RNA polymerase II. Initiation is associated with H3K4 di- and trimethylation and CTD serine 5 phosphorylation (s5pCTD), while H3K36 trimethylation and CTD serine 2 phosphorylation (s2pCTD) correlate with elongation [38]. *In vitro *and *in vivo *approaches were undertaken to address if uH2A affects transcription initiation or elongation.

**Figure 2:**
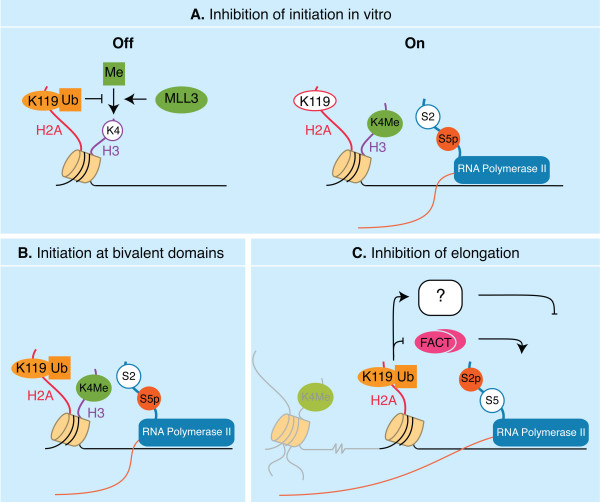
**Models for regulation of transcription by monoubiquitinated H2A**. **A**. uH2A inhibits RNA polymerase II initiation *in vitro *through a crosstalk with H3K4 methylation. uH2A prevents H3K4 methylation by the MLL3 histone methyltransferase. The enzymatic removal of ubiquitin from H2A by USP21 can positively influence H3K4 methylation by the methyltransferase. This would allow H3K4 methylation, which is a prerequisite for initiation of gene transcription. **B**. At bivalent promoters uH2A and H3K4 Me coexist. At these promoters, transcription initiation occurs despite the presence of uH2A. **C**. uH2A might inhibit transcription elongation or the transition from initiation to elongation by preventing association of the FACT elongation factor. In addition, uH2A might regulate elongation by recruiting inhibitory factors and/or affecting higher order chromatin association.

First, uH2A engagement in the initiation step of transcription is supported by *in vitro *studies by Nakagawa and colleagues [25]. GAL4-VP16 driven transcription was assayed on reconstituted chromatin templates using *Drosophila *nuclear extracts as a source of RNA polymerase II. As previously mentioned, uH2A inhibited transcription if the chromatin template was assembled before addition of the RNA polymerase. On similarly reconstituted chromatin templates, the authors showed that uH2A prevents H3K4 di- and trimethylation by the methyltransferase MLL3, and that USP21-mediated deubiquitination could relieve this inhibition (Figure 1B). Importantly, chromatin assembled with a specific mutant of histone H3 (H3K4R), allowed initiation despite the presence of uH2A. Under these conditions, elongation occurred normally, suggesting that uH2A does not inhibit transcription *per se*, but that it might rather act by preventing H3K4 methylation, and thereby transcription initiation (Figure 2A).

*In vivo *support for this conclusion came from the study of Zhu *et al*. on 2A-DUB [27]. Expression of the *PSA *gene is induced upon stimulation of the AR. The authors examined chromatin changes at the *PSA *promoter, upon AR activation, through detailed ChIP experiments. AR activation resulted in a decrease of uH2A accompanied by increased levels of S5pCTD at the promoter and, therefore, increased transcription initiation. All these events were 2A-DUB dependent. Although the levels of H3K4Me were not examined, this study suggests that uH2A limits initiation *in vivo *as well.

Second, the link between uH2A and transcription elongation was examined in two recent papers on H2A E3 ligases, Ring1A/B and 2A-HUB [39,40].

Stock and colleagues examined chromatin changes at genes that are derepressed upon conditional deletion of Ring1A and B and global loss of H2A monoubiquitination in mouse ES cells [39]. The promoters of these genes have been shown to associate with histone marks characteristic of both active (H3K4Me) and inactive (H3K27Me, uH2A) chromatin and are therefore referred to as bivalent [39,41,42], reviewed in [43]. Stock *et al*. showed that in wildtype cells, low-level transcription of the 5' region of the coding region of these genes was detectable. This result indicates that transcription was initiated, despite the presence of Ring1A/B and uH2A (Figure 2B). Indeed, at these promoters, S5pCTD RNA polymerase was present at levels comparable to actively transcribed genes. Given that RNA polymerase II was shown to associate with regions downstream of the promoter upon Ring1A/B deletion, the data support a model in which Ring1B-dependent uH2A hinders transcription at the stage of elongation (Figure 2C).

The involvement of uH2A in transcriptional events downstream of initiation is further supported by the characterization of a novel RING-type E3 ligase for H2A, 2A-HUB [40] (Table 2). Knockdown of 2A-HUB stimulates elongation but not initiation of transcription of one of its target genes, *RANTES*. uH2A is mainly present at the promoter of *RANTES*, as opposed to its distribution over both promoter and exonic regions at bivalent genes. As a consequence, in the case of *RANTES*, uH2A might regulate the transition of initiation to elongation, a process referred to as promoter escape.

In conclusion, it is still unclear at which stage uH2A affects the transcription cycle. It is of note that current data have been obtained in different *in vivo *and *in vitro *systems. For example, uH2A inhibits H3K4 methylation *in vitro*, whereas these marks, by definition, coexist at bivalent promoters in ES cells *in vivo*. This suggest that the strict inhibition of H3K4 methylation by uH2A observed *in vitro *is somehow circumvented at bivalent promoters *in vivo*, allowing initiation. If this interpretation holds true, it would predict that there might be different categories of target genes, regulated by dedicated mechanisms. In addition, we can envisage that, *in vivo*, uH2A may act as a "landing platform" for recruitment of, yet to be isolated, regulatory proteins containing ubiquitin binding domains (Figure 2C). The identification of such proteins will be pivotal to the understanding of uH2A-mediated regulation. Finally, it cannot be excluded that effects of the DUBs and E3 ligases targeting H2A on transcription phases are partially independent of their enzymatic function.

### Histone crosstalks and chromatin dynamics

Work on the DUBs USP21 and 2A-DUB, suggest that uH2A may affect post-translational modifications on other histones, including H3K4 methylation (as discussed before) and H1 phosphorylation [25,27] (Figure 1B and 1C). Such "trans-histone" cross-talk between uH2B and H3 methylation has been well characterized [10]. We have previously discussed how uH2A negatively affects H3K4 methylation. Here we will report on the uH2A-H1 interplay. Also, we will present findings pointing to a distinct mechanism, involving the histone chaperone FACT.

The C-terminal tail of H2A, containing the K119 ubiquitination site, can reach the linker histone H1 in nucleosomes [4]. Immunoprecipitation of nucleosomes containing wt or its ubiquitination mutant, K119R, revealed that H1 preferentially associated with wt H2A, suggesting that ubiquitination of H2A facilitates interaction with H1 [40]. In agreement, this has been shown *in vitro *[44]. H1 can be phosphorylated: a modification linked to enhanced H1 dynamics and chromatin dissociation [45]. Zhou and colleagues reported that a global increase in uH2A, in 2A-DUB knock down cells, was associated with a decrease in phosphorylated H1 [40]. Knockdown of Ring1B had the opposite effect. Altogether, these findings suggest that uH2A hinders H1 eviction from chromatin, which is generally associated with an open chromatin conformation favorable to transcription (Figure 1C).

Finally, a functional link between uH2A and the histone chaperone FACT (FAcilitates Chromatin Transcription) has been proposed [40]. The FACT complex has been shown to facilitate elongation, presumably by removal of H2A/H2B dimers, thereby enabling RNA polymerase II movement through chromatin [46]. Chromatin association of FACT at the *RANTES *gene is stimulated by 2A-HUB knockdown, with concomitant decrease in uH2A. Biochemically, it was shown that FACT associates mostly with non-ubiquitinated H2A. A possible conclusion is that uH2A prevents FACT association. H2A deubiquitination would allow FACT to bind and to promote elongation. More detailed biochemical analysis is required to shed light on the mechanistic implications of uH2A-FACT interplay. An important implication of the 2A-DUB and 2A-HUB studies from the Rosenfeld lab is that ubiquitination of H2A may impact on nucleosome dynamics (as also discussed in the section on the DNA damage response), as well as on higher order chromatin structure.

In summary, the discussed studies highlight a positive role of H2A DUBs in gene expression. In addition, albeit with some redundancy [27-29], it seems that Ubp-M, USP21, USP22 and 2A-DUB participate in the regulation of specific transcriptional programs (Table 1). To understand how DUBs regulate transcription and how specific their activities are, important questions are i) where, along the chromosomes, do these enzymes bind and ii) which genes do they regulate. Gene expression profiles and genome-wide identification of the *in vivo *DNA binding sites of the DUBs (by ChIP on ChIP, Chip-Seq or DamID techniques) will be needed and will require extensive effort in the next years. Because of the difficulty in developing highly specific antibodies to uH2A, an additional challenge will be to map the chromosomal regions containing uH2A. Finally, integration of these data with available genome-wide chromatin association data on histone modifications and crucial chromatin modifiers, including histone E3 ligases, methyltransferases, demethylases and remodeling enzymes, will likely lead to further insight into the intricate cross talk between histone modifications.

## uH2A: a marker for DNA damage?

Genome integrity is maintained by the functional interplay between DNA repair processes and DNA damage checkpoint pathways, responsible for arresting the cell cycle to allow faithful repair [47]. Histone modifications play a crucial role both in DNA damage response (DDR) as well as DNA repair. They can act by i) facilitating DDR signaling or ii) influencing chromatin folding/organization. This second mode is mainly achieved by controlling the binding of effector proteins, among which chromatin remodeling factors, capable of altering histone-DNA contacts [48].

Evidence is accumulating that histone ubiquitination is part of the response to DNA damage. Studies in *S. cerevisiae *suggest a role for ubiquitinated H2B in checkpoint activation upon UV challenge [49] and in the formation of DNA double-strand breaks (DSBs) at some chromosomal loci during meiosis [50]. In mammalian cells, increased monoubiquitination of H2A, H3, and H4 was shown upon UV irradiation [16,21,51]; reviewed in [52]. The recent characterization of two novel histone modifiers, the DUB USP3 and the E3/E2 ligase complex RNF8-Ubc13, supports a broader role of uH2A in genome maintenance [17-19,53-55]. Here we will discuss the involvement of uH2A in the response to ionizing radiation (IR) emerging from these studies.

IR induces the re-localization of DNA damage signaling/repair factors into IRIF (IR-induced nuclear foci). Beside protein accumulation, IRIF reflect chromatin rearrangements and histone post-translational modification at double-stranded DNA breaks (DSBs) [56,57]. Phosphorylation of the histone H2A variant H2AX (γH2AX) by ATM (ataxia telangiectasia mutated), ATR (ATM and Rad3-related), and DNA-PK (DNA-dependent protein kinase) checkpoint kinases is an early event in response to DNA damage and represents the most robust histone modification upon IR [47]. γH2AX is instrumental for efficient accumulation and retention of several mediators/repair factors, including MDC1, BRCA1, 53BP1 and ATM, at the chromatin surrounding the lesion [58,59].

Several ubiquitination events take place at DSBs repair sites, as illustrated by the local accumulation of ubiquitinated substrates at IRIF [53,60-62]. However, only recently, immunofluorescence studies, with an anti-uH2A antibody, detected accumulation of uH2A at γH2AX-containing nuclear foci early upon global IR as well as local, laser-mediated microirradiation [17,19]. Which species of ubiquitinated H2A accumulate at DSBs? Immunopurification of endogenous or ectopically expressed H2A or H2AX revealed an increase in oligo and poly-ubiquitinated species in cells exposed to high IR doses [18-20,53]. Huen *et al*. showed that H2AX was predominantly di-ubiquitinated upon IR. Notably, H2AX di-ubiquitination was dependent on its S139 phosphorylation site, suggesting that phosphorylation is a prerequisite for ubiquitination [18]. As to the ubiquitination site, it is still unclear whether in addition to the canonical lysine 119, ubiquitination of other lysines may participate in the DSB response [18,20]. These studies suggest that IR-induced ubiquitin marks on H2A/H2AX comprise a variety of ubiquitinated species, which differ from the steady state uH2A.

### How is the histone ubiquitin mark set during the IR-response?

**Figure 3:**
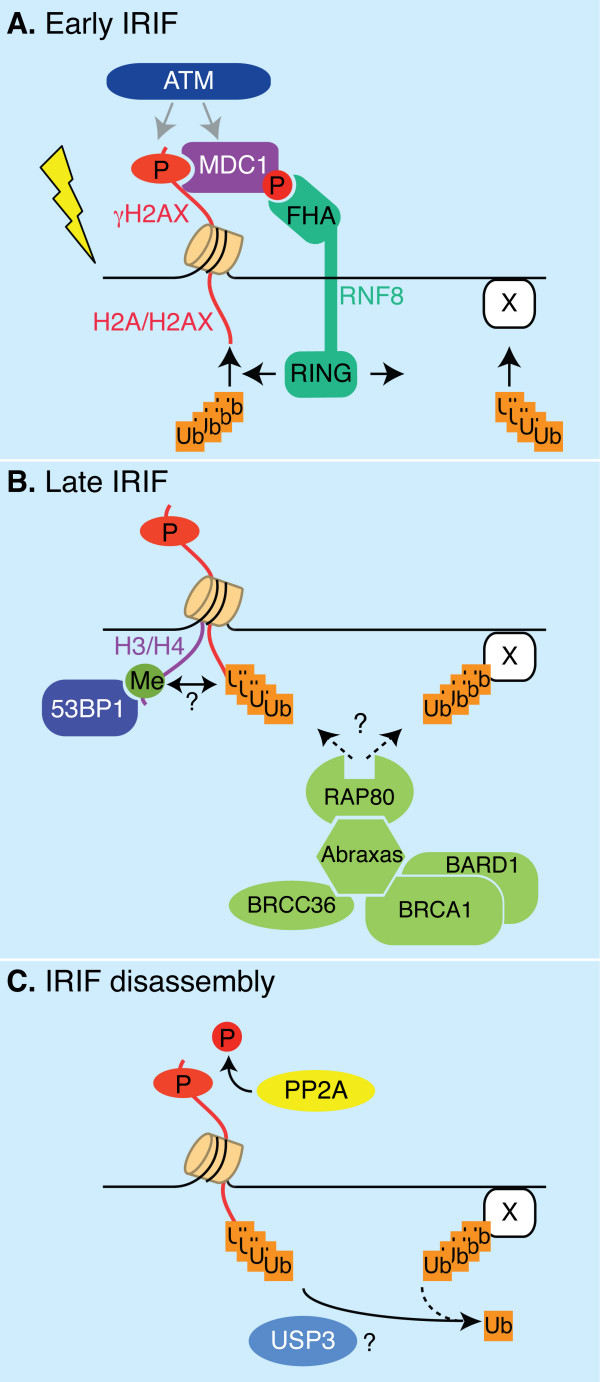
**Signaling network at Ionizing Radiation Induced Nuclear Foci (IRIF)**. **A**. Early after IR, damaged DNA triggers ATM activation, which consequently phosphorylates H2AX near the break sites and MDC1. RNF8 is recruited to IRIF through binding to phosphorylated MDC1. RNF8 locally ubiquitinates H2A, H2AX and possibly other yet unidentified proteins ('X'). **B**. RNF8-dependent ubiquitin conjugation is required for recruitment of 53BP1 and BRCA1. BRCA1 is present in a protein complex containing BARD1, Abraxas/CCDC98, BRCC36 and RAP80. Ubiquitin binding by RAP80 is required for BRCA1 recruitment. However, the nature of the ubiquitinated substrate RAP80 binds to (histones or other proteins), is still unclear. In addition to BRCA1, ubiquitination allows recruitment of 53BP1 (which binds to methylated H3/H4) through a yet unknown mechanism. **C**. IRIF disassembly may involve dephosphorylation of γH2AX by PP2A. The DUB USP3 is a candidate to remove conjugated ubiquitin at IRIF.

Four independent studies recently identified RNF8 as the E3 ligase responsible for H2A/H2AX ubiquitination in the response to DSBs [18,19,54,55]. RNF8 rapidly accumulates at DSBs upon IR, concomitantly with early IRIF markers, namely γH2AX, ATM, the MRN complex, and the mediator protein MDC1 [19]. The presence of a phosphothreonyl-binding FHA domain together with a RING finger domain enable RNF8 to link phosphorylation with ubiquitination signaling at IRIFs (Figure 1D). The data presented by the four laboratories are consistent with a signaling cascade starting from phosphorylation of H2AX by ATM (Figure 3). This step is well known and allows direct recruitment of MDC1 and its subsequent phosphorylation by ATM [59]. Through its FHA domain, RNF8 can in turn bind to phospho-MDC1 at DSBs, where it catalyzes polyubiquitination events, among which H2A and H2AX ubiquitination (Figure 3A). At IRIF, RNF8 likely acts in concert with the ubiquitin conjugating enzyme Ubc13, a previously reported interactor of RNF8 [18,53-55,63]. At last, RNF8-Ubc13-dependent ubiquitination is required for recruitment and retention of BRCA1 and 53BP1 at DSBs [18,19,54,55] (Figure 3B). Besides histone ubiquitination, ubiquitin signaling at IRIF likely comprises a complex variety of ubiquitination events, as also suggested by the partial requirement of a second E3 ligase activity, BRCA1, for efficient ubiquitin accumulation [60,62]. These may include amplification of the ubiquitination signal on histones or other substrates, as well as autoubiquitination of the E3 enzymes as autoregulatory mechanism [64,65]. In agreement, both RNF8 and BRCA1 can promote auto-ubiquitination and ubiquitination of histones *in vitro *[19,63,66,67].

These studies revealed an exciting link between ATM signaling and ubiquitination of histone H2A and H2AX at double strand breaks. If uH2A is directly involved in recruitment of DDR factors at IRIF remains to be established.

### Mechanisms of uH2A-mediated DDR

The ubiquitin-interacting-motif (UIM)-containing protein Rap80 is a good candidate for fulfilling a ubiquitin receptor function at IRIF. Rap80 was initially shown to recruit the Rap80-ccd98/abraxas-BRCA1 complex to ubiquitin-conjugates at DSBs [61,68,69] (Figure 3B). More recently, it was demonstrated that accumulation of Rap80 at IRIF is dependent on functional RNF8-Ubc13 proteins [18,19,54,55]. *In vitro *and *in vivo *Rap80 displays preferential binding to lysine63-isopeptide-linked tetraubiquitin polymers, and to a lesser extent to K6-linked chains [61]. Importantly, Rap80 ubiquitin binding properties fit well with the findings that i) chain formation through lysine 63 has been previously shown to regulate, among others, DNA repair processes [60], ii) ubiquitin foci at IRIF form most efficiently through K63-linked polyubiquitination [61] and iii) Ubc13 is the only E2 able to catalyze polyubiquitination through K63 [70], and it is an E2 partner for RNF8 [63]. Although these evidences are consistent with Rap80 linking RNF8-Ubc13 ubiquitylation at IRIF to the DDR mediator BRCA1, studies addressing the potential direct binding of Rap80 to ubiquitinated H2A/H2AX are still needed.

It is important to note that other mechanisms may exist through which H2A ubiquitination could affect DDR. One plausible possibility is that uH2A/H2AX may facilitate a more global alteration of the chromatin, known to occur at DSBs [57,71], allowing exposure of other histone marks. This mechanism might be relevant for 53BP1 relocalization to IRIF, as suggested by the findings that 53BP1 binds to methylated histones [72,73] and that its recruitment is independent from Rap80 [18,19,55,68]. Interestingly, Ikura and colleagues reported enhanced mobility of H2AX at sites of microirradiation and a rapid release of polyubiquitinated H2AX from the chromatin [20]. H2AX ubiquitination and histone release was dependent on TIP60 and on its acetylation target, H2AX K5 (Figure 1D). Drosophila Tip60 can acetylate H2Av (the Drosophila H2AX) and promotes its exchange with unphosphorylated H2Av [74]. This work suggests a mechanism by which ubiquitination might promote histone dynamics/removal.

### Removal of uH2A mark at DSBs: a role for USP3?

The mechanisms by which the chromatin post-translational modifications are cleared, the protein assemblies are disassembled, and the checkpoint is turned off after completion of repair is an intriguing open question. Work form our group recently showed that USP3, previously identified as a DUB [75], displays deubiquitination activity both towards uH2A as well as uH2B *in vivo *[17]. USP3 appears to be engaged in DDR in two majors ways: in the response to IR and during normal S-phase progression (as discussed below). Upon IR, IRIF containing uH2A, ubiquitin, and γH2AX persist in USP3 knockdown cells [17]. Previously published results demonstrated that dephosphorylation and removal of H2AX is needed for efficient repair and resumption of the cell cycle [76-78]. In agreement, USP3 knock down cells are significantly delayed in G2/M transition. Together, these data suggest that removal of ubiquitination marks at the chromatin surrounding DSBs is necessary to coordinate IRIF disassembly, γH2AX dephosphorylation and cell cycle recovery (Figure 3C). Unlike in *S. cerevisiae *[77,78], dephosphorylation of H2AX by the phosphatase PP2A is thought to occur on the chromatin, given that this enzyme localizes to IRIF [76]. If and how USP3 crosstalks with PP2A, for example by affecting recruitment or activity of the latter, is currently unknown. Although USP3 seems to favor deubiquitination of substrates (among which uH2A) at IRIF, we could not detect local accumulation of USP3 at those foci (unpublished results). This may be a consequence of the fact that USP3 rapidly releases its chromatin substrate upon catalysis as determined by Fluorescence recovery after photobleaching (FRAP) and co-immunoprecipitation [17]. Also, it is possible that USP3 DUB activity may be required 'off the chromatin" or may be indirect. Isolation of USP3 interacting partners and potential additional substrates, as well as addressing if and how USP3 is regulated, is needed to gain a better understanding of the molecular mechanism of USP3-mediated DDR.

A JAMM domain containing DUB, BRCC36, may also play a role at DSBs: it is part of the BRCA1 A complex (together with RAP80 and Abraxas), localizes to IRIF and positively regulates BRCA1/BARD1 E3 ligase [55,61,79,80]. However, BRCC36 role at IRIF is not clear and its activity towards ubiquitinated histones has not been investigated.

In summary, the discovery of novel E3 ligase (RNF8) and DUB activities (USP3) for H2A provides us with valuable tools to address ubiquitin-mediated signaling at the chromatin. We can envision several experimental approaches towards the elucidation of the molecular mechanism(s) of uH2A/uH2AX-mediated DDR. These include: i) biochemical characterization of the E3 (RNF8, BRCA1) and DUB activities (USP3, others?) on nucleosomes; ii) identification of the IR-induced ubiquitination site(s) on H2A/H2AX; iii) isolation of uH2A/uH2AX binding proteins; iv) definition of the extent of the ubiquitin mark around the DSB. Also, a proteomic approach towards the characterization of the complex mixture of ubiquitinated proteins and ubiquitin receptors at the chromatin will potentially reveal novel players in DDR.

## uH2A and cell cycle

Histone modifications play a pivotal role in the regulation of chromatin packaging during cell cycle progression. During G0-G1/S transition, repressive histone marks need to be removed to allow expression of S-phase genes [30,81]. Genome-wide chromatin rearrangements occur in S-phase, in order to provide accessibility to the replication machinery and to restore the epigenetic landscape on the newly synthesized DNA (reviewed in [82]). Finally, chromatin condensation and decondensation is needed during mitosis and cell division (reviewed in [5]).

As most of histone marks, ubiquitination is cell cycle regulated. uH2A was initially reported to be reduced in resting (G0) and differentiated cells [22]. Later, Vassilev *et al*. described a monoclonal antibody able to recognize uH2A. Using this antibody, the authors reported that non-proliferating cells displayed lower levels of uH2A than their proliferating counterparts. In addition, partial co-localization of uH2A with PCNA replication foci was detected, suggesting a role for uH2A in cellular proliferation/DNA replication [83]. Interestingly, a link between uH2A and (aberrant) cell growth is emerging also *in vivo*. Dynamic changes in uH2A were observed in a liver regeneration model and in primary prostate tumors compared to normal tissues [25,27]. In proliferating cells, uH2A is present throughout the cell cycle, but it is down-regulated during G2/M transition and is not present on condensed chromosomes [23,24].

### H2A-DUBs: specific roles in cell cycle regulation?

Among the DUBs targeting H2A, USP22, Ubp-M and USP3 have been connected to cell cycle progression. Consistently, knockdown of these enzymes resulted in growth impairment in different human cell lines, albeit in different ways: i) upon shRNA-mediated knock-down of USP22, both p53 deficient H1299 cells and normal human fibroblast accumulated in G1 [28]; ii) USP3-depleted U2OS cells showed a strong delay in S-phase progression and a low mitotic index [17]; iii) stable Ubp-M knockdown in HeLa cells resulted in a decrease in the proportion of cells in G2/M [24]. The potential impact of the two other H2A DUBs, 2A-DUB and USP21, on cell cycle progression has not been investigated yet [25,27].

As discussed before, uH2A is functionally linked to transcription regulation and DNA damage signaling pathways. Does uH2A affect cell proliferation through transcriptional regulation of cell cycle genes or DDR activation, or through direct mechanism(s)? The data suggest that both indirect and direct mechanisms may be relevant.

First, USP22 has been characterized as a transcriptional co-activator in the context of the SAGA complex (see also the previous paragraph on transcription) [28,29]. Consequently, it is possible that the USP22 inhibitory effect on G1/S transition is dependent on its transcriptional activity. In addition, USP22 is required for Myc-driven transcription and transformation. Given that USP22, as well its Drosophila homolog Nonstop, appear to regulate a large subset of genes, gene expression analysis and genetic studies will be required to identify the key USP22-target genes involved in cell cycle progression [28,29,35].

Second, data by our group point to a distinct mechanism by which USP3 may affect cell cycle. S-phase delay in USP3-KD cells is accompanied by spontaneous γH2AX foci, accumulation of DNA breaks and full activation of an ATM- and ATR-mediated checkpoint response [17]. Specific phenotypes were observed upon USP3 KD, including i) reduced ability to incorporate BrdU, witnessing impaired DNA synthesis, ii) the presence of single-stranded DNA coated by replication protein A (RPA), iii) activation of the ATR-Chk1 pathway. These features are consistent with USP3 KD cells undergoing replication stress and suggest that S-phase delay and G2/M arrest are intimately linked to DDR activation in these cells. Wheter USP3 acts directly affecting the DNA replication process/machinery, or rather through uH2A-mediated DDR signaling, is presently the subject of investigation. Persistence of γH2AX and uH2A DNA damage foci in USP3 KD cells upon thymidine-induced replication stress, as well as upon exogenous DNA damage (as discussed before), supports the participation of USP3 in ubiquitin (uH2A)-mediated signaling (F. Nicassio and E. Citterio unpublished results). Finally, the observation that γH2AX foci, in USP3-depleted cells, coincide with the onset of S-phase suggests that DNA replication is required for DDR activation (F. Nicassio and E. Citterio unpublished results). Investigating how USP3 influences replication dynamics will possibly contribute to the understanding of USP3/uH2A role at replication forks and its functional interplay with the S-phase checkpoint.

Third, H2A is globally de-ubiquitinated in the G2/M transition, which correlates with phosphorylation of H3 at S10, a hallmark of mitosis [24]. Interestingly, Joo *et al*. showed that Ubp-M is required for mitotic H2A de-ubiquitination and that knockdown of UbpM results in G2/M delay. In addition, *in vitro *experiments using reconstituted nucleosomes reveal that uH2A inhibits H3 phosphorylation on serine 10 by AuroraB, at least in part by preventing the binding of the kinase to nucleosomes (Figure 1E). De-ubiquitination of H2A by UbpM relieves the inhibition of AuroraB. Since H3S10 phosphorylation by AuroraB is needed for chromosome condensation, these *in vitro *results put forward the exciting possibility that Ubp-M-mediated H2A deubiquitination may affect G2/M transition through a direct mechanism. Further investigation will reveal if this is true *in vivo*.

Intriguingly, despite targeting the same substrate, H2A DUBs display specificity in their roles in cell cycle regulation. It is unknown whether this specificity is due to additional protein targets, besides H2A, or to other mechanisms. Therefore, it will be interesting to assess if different H2A DUBs can rescue each other defects in cell cycle progression.

Finally, the H2A E3 ligases Ring1B and RNF8 are clearly implicated in cell proliferation. Work by different groups support a functional role for RNF8 in mitosis, showing its requirement for mitotic exit [84,85]. Whether the effects of RNF8 on mitosis are dependent on ubiquitination of H2A remains to be investigated. As to Ring1B, its deletion in mice is associated with gastrulation arrest and embryonic lethality [86]. Given that Ring1B deletion strongly impact on expression of its target genes, it is difficult to distinguish between direct and indirect effects of Ring1B on cell cycle progression [86,87]. Knockdown of Ring1B results in cell cycle arrest in U2OS cells [11]. It will be interesting to analyze this phenotype in more detail to assess which phase of the cell cycle is affected.

## Concluding remarks

Overall, the discussed data show that uH2A impacts on several important aspects of cellular physiology. Although recent work provides crucial insights, we are just starting to decipher the role of uH2A. Intriguingly, despite the common substrate, individual H2A DUBs seem to exhibit distinct functional roles. In this regard, we will put forward key questions concerning regulation of activity, recruitment and substrate specificity, whose addressing is predicted to greatly advance our knowledge of how DUBs impact on the pleotropic function of H2A.

How is the activity of the H2A DUBs regulated? Interaction with regulatory, non catalytic protein subunits and posttranslational modifications have been shown to regulate DUB activity, including activity of USP7, USP1 and USP28 [88-93]. Similar mechanisms may apply to the H2A DUBs. USP22 displays *in vitro *H2A DUB activity only in the context of SAGA, suggesting that other subunits of the complex are important for the regulation of its activity [27,29]. Phosphorylation of UbpM (USP16) and mono-ubiquitination of 2A-DUB have been reported [24,27,33]. However, the functional significance of these observations remains to be elucidated.

How are H2A DUBs recruited to specific chromosomal loci? As discussed before, in the case of USP22 and 2A-DUB, local recruitment to their respective target genes has been shown to depend on components of the protein complexes they reside in (SAGA for USP22), as well as on specific transcription factors [27-29]. The finding that two DUBs, 2A-DUB and USP22 associate with HATs suggests an important functional interplay between these classes of enzymes. Biochemical purification of the other DUBs (complexes) has yet to be performed.

Do H2A DUBs target additional substrates for deubiquitination? USP3 and USP22 are capable of deubiquitinating H2B, in addition to H2A [17,28,29,35]. Moreover, USP22 may be required for deubiquitination of additional non-histone proteins, as suggested by accumulation of other ubiquitinated proteins upon loss-of-function of its Drosophila homolog [94]. The ability to target more than one substrate is not unprecedented among DUBs [90,93]. The identification of putative additional key substrates is essential for a better understanding of the function of the H2A DUBs.

Finally, multiple observations suggest that H2A (de)ubiquitination influences chromatin dynamics, in part through the action of histone chaperones such as FACT and Tip60 [20,27,40]. This may prove a common mode to facilitate chromatin reorganization during transcription, DDR and DNA replication, explaining the diversity of processes that H2A DUBs and uH2A play a role in. It will be important to examine the effect of ubiquitination of H2A on histone mobility and to define how uH2A impacts on the activities of chromatin remodeling complexes/histone chaperones. The identification of the DUBs and E3 ligases described in this review, as well as potential novel ones will undoubtedly greatly facilitate such analysis.

## Competing interests

The authors declare that they have no competing interests.

## Authors' contributions

JHAV, FN and EC wrote the paper. JHAV designed the figures. All authors contributed to the ideas elaborated in the paper and read and approved the final manuscript.
